# From Grape By-Products to Enriched Yogurt Containing Pomace Extract Loaded in Nanotechnological Nutriosomes Tailored for Promoting Gastro-Intestinal Wellness

**DOI:** 10.3390/antiox12061285

**Published:** 2023-06-15

**Authors:** Ines Castangia, Federica Fulgheri, Francisco Javier Leyva-Jimenez, Maria Elena Alañón, Maria de la Luz Cádiz-Gurrea, Francesca Marongiu, Maria Cristina Meloni, Matteo Aroffu, Matteo Perra, Mohamad Allaw, Rita Abi Rached, Rodrigo Oliver-Simancas, Elvira Escribano Ferrer, Fabiano Asunis, Maria Letizia Manca, Maria Manconi

**Affiliations:** 1 Department of Life and Environmental Sciences, University of Cagliari, University Campus, Pad. A, S.P. Monserrato-Sestu Km 0.700, 09042 Monserrato, CA, Italy; ines.castangia@unica.it (I.C.); federica.fulgheri92@unica.it (F.F.); fmarongiu@unica.it (F.M.); mariacristina.meloni@unica.it (M.C.M.); matteo.aroffu@unica.it (M.A.); allaw.mohamad.22@gmail.com (M.A.); r.abirached@studenti.unica.it (R.A.R.); manconi@unica.it (M.M.); 2 Regional Institute for Applied Scientific Research (IRICA), University of Castilla-La Mancha, Avda. Camilo José Cela 10, 13071 Ciudad Real, Spain; javier.leyva@uclm.es (F.J.L.-J.); mariaelena.alanon@uclm.es (M.E.A.); rodrigo.oliver@uclm.es (R.O.-S.); 3 Department of Analytical Chemistry and Food Science and Technology, University of Castilla-La Mancha, Ronda de Calatrava 7, 13071 Ciudad Real, Spain; 4 Department of Analytical Chemistry, University of Granada, Campus Fuentenueva S/N, 18071 Granada, Spain; mluzcadiz@ugr.es; 5 Biomedical and Tissue Engineering Laboratory, Fundación de Investigación Hospital General Universitario, 46022 Valencia, Spain; 6 Biopharmaceutics and Pharmacokinetics Unit, Institute for Nanoscience and Nanotechnology, University of Barcelona, 08028 Barcelona, Spain; eescribano@ub.edu; 7 Department of Civil, Environmental Engineering and Architecture (DICAAR), University of Cagliari, Piazza D’Armi 1, 09123 Cagliari, Italy; fabiano.asunis@unica.it

**Keywords:** dextrin fibers, gelatin, antioxidant activity, enriched yogurt, gut protection

## Abstract

Grape pomace is the main by-product generated during the winemaking process; since it is still rich in bioactive molecules, especially phenolic compounds with high antioxidant power, its transformation in beneficial and health-promoting foods is an innovative challenge to extend the grape life cycle. Hence, in this work, the phytochemicals still contained in the grape pomace were recovered by an enhanced ultrasound assisted extraction. The extract was incorporated in liposomes prepared with soy lecithin and in nutriosomes obtained combining soy lecithin and Nutriose FM06^®^, which were further enriched with gelatin (gelatin-liposomes and gelatin-nutriosomes) to increase the samples’ stability in modulated pH values, as they were designed for yogurt fortification. The vesicles were sized ~100 nm, homogeneously dispersed (polydispersity index < 0.2) and maintained their characteristics when dispersed in fluids at different pH values (6.75, 1.20 and 7.00), simulating salivary, gastric and intestinal environments. The extract loaded vesicles were biocompatible and effectively protected Caco-2 cells against oxidative stress caused by hydrogen peroxide, to a better extent than the free extract in dispersion. The structural integrity of gelatin-nutriosomes, after dilution with milk whey was confirmed, and the addition of vesicles to the yogurt did not modify its appearance. The results pointed out the promising suitability of vesicles loading the phytocomplex obtained from the grape by-product to enrich the yogurt, offering a new and easy strategy for healthy and nutritional food development.

## 1. Introduction

In recent years, according to the International Organization of Vine and Wine, world wine consumption has increased significantly [1]. As a result, the residues generated during the vinification process have received much interest, estimating that pomace represents ~25% of the processed grapes. Currently, they are treated as waste or as a source of ethanol or energy and induce relevant environmental and economic impacts [2]. Instead, they are a precious source of bioactive molecules such as flavonoids, tannins or phenolics that may act as antioxidants, anti-inflammatory and antimicrobial agents, for humans and animals [3]. In a previous study, pomace of Carignano grapes cultivated in Sulcis, in the southwestern region of Sardinia (Italy), was found to be rich in catechin, quercetin, fisetin and gallic acid, which are known for their antioxidant and beneficial activities [4,5,6]. These molecules are contained in grape pomace as complexes, mixed with sugars, fibers and polymers; need to be separated using advanced methodologies, to avoid economic and energetic dissipative steps; and reach high yields [7]. Extraction is the first step on the pathway to the grape pomace valorization and the final composition and biological activity of extracts depends on the procedure used to obtain them [8]. The second step to their use in large scale products is the improvement of their in vivo bioavailability since the majority of them are poorly water soluble and highly unstable, especially to light or heat, and their beneficial activities often decrease during digestion, due to pH variations and the action of enzymes such as pepsin and pancreatin [9,10]. To overcome these drawbacks and facilitate their addition to foods, the extracts could be loaded in nanocarriers suitable for oral administration and in this form, they could be added to several foods [11]. Indeed, the contemporary industrial production of foods involves the addition of functional ingredients to tailor flavor, color, texture and preservation or to improve the beneficial properties. To this purpose, the latest trend foresees the inclusion of bioactive molecules in nanocarriers [12]. Among the different nanocarriers suitable for food fortification, phospholipid vesicles are promising since they are mainly formed by phospholipids, which naturally occur in the human body as the main constituents of the membrane of cells. 

Usually, all extracts obtained from grape are considered suitable for intestinal protection and are able to improve food quality [13]. Their beneficial effects could be maximized when loaded in phospholipid vesicles, due to their role as effective carriers to protect the payloads and improve their local and systemic efficacy [7]. Recently, it has been proven that the addition of Nutriose FM06^®^ to phospholipid vesicles achieved the formation of optimal carriers, so-called nutriosomes, ideal for the oral administration of natural bioactive molecules or phytocomplexes and their delivery to the intestinal tract [14,15]. Nutriose FM06^®^ is a water-soluble, branched dextrin with a high fiber content obtained from corn, formed by dextrin linked with digestible α-1,6 glycosidic linkages and non-digestible α-1,2 and α-1,3 glycosidic linkages. Thanks to this chemical structure, only 10 to 15% of the fiber is absorbed in the stomach and small intestine, the remaining being progressively fermented in the colon, giving a prebiotic effect [16]. Due to its unique gelling properties, gelatin is one of the most popular protein biopolymers, widely used in food, pharmaceutical and cosmetic fields [6,17]. Previous results underlined that the modification of nutriosomes with the addition of gelatin or other polymers can further improve their performances [18]. In a recent work, Perra et al. (2022) also demonstrated that gelatin can be effectively added to liposomes and nutriosomes and they improve the effect of the grape extract on the stimulated proliferation of *Limosilactobacillus reuteri*, even at lower concentrations [6]. 

Considering previous results and aiming at enriching a yogurt with grape pomace extract loaded in phospholipid vesicles, in the present study, the extract obtained from Carignano grape pomace was loaded in liposomes, nutriosomes, gelatin-liposomes and gelatin-nutriosomes. The main physico-chemical characteristics and stability of vesicles at different pH values simulating the saliva (6.75), stomach (1.20) and intestine (7.00) were measured. Their biocompatibility in Caco-2 cells, along with their ability to protect cells from damage caused by oxidative stress, were assessed. Finally, vesicle dispersions were added to the yogurt and their main properties, appearance, stability and viscosity were evaluated. 

## 2. Materials and Methods

### 2.1. Materials

Lecithin was purchased from Galeno (Potenza, Italy). Gelatin and all other products of analytical grade were purchased from Sigma-Aldrich (Milan, Italy). Nutriose FM06^®^, a soluble dextrin from maize, was kindly provided by Roquette (Lestrem Cedex, Beinheim France). The cell medium, fetal bovine serum, penicillin and streptomycin, and all the other reagents for cell studies, were purchased from Thermo-Fisher Scientific Inc. (Waltham, MA, USA). 

### 2.2. Plant Material and Extraction Method

The extract was obtained from skins of Carignano grape pomace, kindly provided by Cantina Santadi (Sardinia, Italy). The skins were manually separated from the seeds, and 50 g of the matrix was suspended in 2 L of distilled water and kept in constant agitation at 25 °C for 24 h. Then, the sample was squeezed and filtered to remove the solvent, freeze-dried, grinded into a fine powder and stored under vacuum and in the dark at 25 °C until solid–liquid extraction. Briefly, 30 g of the grape skins’ powder was suspended in 970 mL of a mixture of ethanol:water (70:30 *v*/*v*, density 0.88556 g/mL). The suspension was shaken in the dark at 25 °C for 48 h. At scheduled times (0, 1, 2, 3, 4, 6, 8, 24 h), samples were sonicated for 1000 s (200 cycles, 5 on, 5 off, 15 μm of probe amplitude) by using a high-intensity ultrasonic disintegrator (Soniprep 150, MSE Crowley, London, UK) to enhance extraction [19]. At the end of the extraction process, the dispersion was centrifuged to separate the solid and liquid phases and the ethanol was eliminated from the extractive solution through low-pressure evaporation in rotavapor (Rotavapor RII, BÜCHI Labortechnik AG, Flawil, Switzerland) coupled to a vacuum pump (Vacuum Pump V-700, BÜCHI Labortechnik AG, Flawil, Switzerland). Water was subsequently removed by freeze-drying, thus obtaining a hygroscopic purple powder that was maintained in the dark and under vacuum until use [19].

### 2.3. Determination of Total Phenolic Content and Antioxidant Activity

The total phenolic content of the skin extract was measured according to the Folin-Ciocalteu colorimetric assay using a UV spectrophotometer (Lambda 25, PerkinElmer, Waltham, MA, USA). The ethanolic extract solution (100 μL, 1 mg/mL), the Folin-Ciocalteu reagent (100 μL) and the sodium carbonate aqueous solution (800 µL, 20% *w*/*v*) were mixed, and the absorbance was read at 765 nm after 30 min of incubation in the dark at 25 °C. The total phenolic content was calculated using a calibration curve obtained by plotting the absorbance as a function of the concentration of gallic acid (0–0.100 mg/mL), and it was expressed as mg of gallic acid equivalent/g of dry extract. The experiments were performed in triplicate.

The antioxidant activity of the extract was assessed by measuring its ability to scavenge the DPPH (2,2-diphenyl-1-picrylhydrazyl) radicals. The ethanolic solution of the extract (20 μL, 1 mg/mL) was mixed with 1980 μL of the DPPH methanolic solution (40 µg/mL), and incubated for 30 min at room temperature in the dark. Then, the absorbance was measured at 517 nm against blank.

The antioxidant activity (AA) was calculated according to the following formula [20]:

(1) $$AA\%=\frac{ABS_{DPPH}-ABS_{sample}}{ABS_{DPPH}}\times 100$$

A calibration curve using trolox (6-hydroxy-2,5,7,8-tetramethylchroman-2-carboxylic acid) at different concentrations (0–0.010 mg/mL) was built and used as a reference, and the antioxidant activity was expressed as mg of trolox equivalent/g of dry extract. All the experiments were performed in triplicate.

### 2.4. Vesicle Preparation 

Lecithin (240 mg/mL) and the grape pomace extract (40 mg/mL) were weighed in a glass vial and hydrated with 2 mL of bi-distilled water to obtain liposomes. Nutriose FM06^®^ (150 mg/mL) was mixed with the lipid and the extract to obtain nutriosomes. Gelatin-liposomes and gelatin-nutriosomes were prepared adding gelatin (10 mg/mL) to liposomes and nutriosomes. The dispersions were sonicated (10 + 10 + 10 cycles, 5 s on and 2 s off, 13 µ of probe amplitude, allowing the sample to cool between each sonication), using a Soniprep 150 ultrasonic disintegrator (MSE Crowley, London, UK). Empty vesicles were also prepared without the grape pomace extract and used as comparison.

### 2.5. Vesicle Characterization 

The average diameter and polydispersity index of the vesicles were determined by photon correlation spectroscopy using a Zetasizer Ultra (Malvern Instruments, Worcestershire, UK). The Zetasizer Ultra was used as well to detect the surface charge of vesicles (zeta potential) measuring their electrophoretic mobility in dispersion by the mixed-mode measurement-phase analysis (M3-PALS). Each sample was diluted with water (1:100) to be optically clear and to avoid the attenuation of the laser beam by the particles along with the reduction of scattered light that can be detected [21].

The entrapment efficiency was calculated as the percentage of antioxidant activity of vesicle dispersions measured before and after their purification by dialysis from the unentrapped extract. The vesicle dispersions (1 mL) were loaded into the dialysis tube (Spectra/Por^®^ membranes, 12–14 kDa MW cut-off, 3 nm pore size; Spectrum Laboratories Inc., DG Breda, The Netherlands) and maintained at room temperature in water (1 L), constantly stirred for 2 h, refreshing the water after 1 h. The antioxidant activity of dispersions before and after the dialysis was measured using the DPPH colorimetric assay, as reported above (see Section 2.3).

### 2.6. Vesicle Behavior at Modulating pH

A saliva simulating solution was prepared by dissolving 2.38 g of sodium phosphate dibasic, 0.19 g of potassium dihydrogen phosphate and 8 g of sodium chloride in 1 L of water. The pH was adjusted to 6.75 with a diluted solution of phosphoric acid. The vesicles were diluted (1:1000) with artificial saliva and maintained at 37 °C for 10 min. The mean diameter, polydispersity index and zeta potential of the vesicles were measured at the end of the incubation period.

Acidic medium simulating gastric fluid (pH 1.20) was prepared dissolving 1.75 g of sodium chloride in 94 mL of water and adding 6 mL of 1 M hydrochloric acid according to USP XXIV (US. Pharmacopeia XXIV) [22].

Neutral medium simulating intestinal fluid (pH 7.00) was prepared dissolving 7.26 g of disodium hydrogen phosphate, 3.56 g of sodium dihydrogen phosphate and 17.54 g of sodium chloride in 1 L of water and sodium chloride (0.3 M) [23].

The average diameter, polydispersity index and zeta potential of vesicles were measured after incubation in the medium at pH 1.20 for 2 h and at pH 7.00 for 6 h at 37 °C.

### 2.7. Biocompatibility of Vesicles

Human colon adenocarcinoma (Caco-2) cells were grown as monolayers in 75 cm^2^ flasks incubated at 37 °C in 100% humidity and 5% CO_2_. Dulbecco’s Modified Eagle Medium (DMEM) with high glucose and L-glutamine, supplemented with 10% of fetal bovine serum and 1% of penicillin-streptomycin (10,000 units/mL of penicillin and 10,000 µg/mL of streptomycin), was used as a growth medium. To ensure their complete differentiation, cells were cultured for at least 10 days and cell division arrested for at least 20 days, and finally trypsinized to be seeded into 96-well plates at a density of 7.5 × 10^3^ cells/well [24]. After 24 h of incubation, the cells were treated with the extract in an aqueous dispersion or loaded in vesicles properly diluted with DMEM to achieve the desired extract concentrations (40, 4 and 0.4 μg/mL). After 48 h of incubation, MTT [3(4,5-dimethylthiazolyl-2)-2, 5-diphenyltetrazolium bromide] (100 μL, 0.5 mg/mL final concentration) was added to each well. After 3 h, the formed formazan crystals were dissolved with dimethyl sulfoxide, and the absorbance was measured at 570 nm with a microplate reader (Synergy 4 Reader, BioTek Instruments, AHSI S.p.A, Bernareggio, Italy). 

To confirm the reliability of the obtained results, the MTT test has been also performed in cell-free plates, using the extract in the dispersion. In this way, we confirmed that the grape extract was not capable of reducing MTT to purple formazan. The results are in agreement with a previous study, which reports that only uncoupler molecules such as rottlerin can interfere with MTT test [25].

The experiments were repeated at least three times, each time in triplicate. Results are reported as a percentage of cell viability in comparison with untreated control cells (100% viability). 

### 2.8. Protective Effect of Vesicles against Damages Induced in Cells by Oxidative Stress

Caco-2 cells were seeded into 96-well plates at a density of 7.5 × 10^3^ cells/well. After 24 h of incubation, the cells were stressed with hydrogen peroxide (30% diluted 1:40,000) and simultaneously treated with the grape extract in aqueous dispersion or loaded in vesicles properly diluted to achieve the desired concentration (4 μg/mL of extract). The cells stressed with hydrogen peroxide only were used as negative control, while untreated cells (100% of viability) were used as a positive control. After 4 h of incubation, the cells were washed with phosphate buffered saline, pH 7.4 (PBS), and their viability was measured by the MTT assay (Section 2.9).

### 2.9. Stability of Vesicles in Milk Whey

Milk whey was obtained as a supernatant after yogurt centrifugation at 4000 rpm and was stored at 4 °C until use. The acidity, the mean size and the polydispersity index of whey particles in dispersion, such as globular proteins and fat micelles, were measured. Vesicle dispersions were mixed with milk whey reaching different concentrations (5, 10 and 20% *v*/*v*) and their average diameter, polydispersity index and zeta potential at 1 h and 24 h of incubation at 4 °C were measured. 

### 2.10. Measurements of Viscosity

Viscosity studies have been carried out using a Brookfield Programmable LVDV-II + Viscometer (AMETEK GB LTD T/A Brookfield Technical Centre, Essex, Harlow, UK), connected to an FE2 HAAKE thermostatic bath. The viscosity of yogurt containing different concentration of vesicles (5, 10 and 20% *v*/*v*) at given shear rates and a controlled temperature (25 ± 2 °C) has been measured. The rotation speed (0.2 RPM, revolutions per minute) was held constant for 30 s, and the viscosity (mPas) as a function of Torque (torsion force expressed as a percentage), shear rate (1/s) and shear stress (dyne/cm^2^) was measured. All measurements were performed in triplicate.

### 2.11. Statistical Analysis of Data

Results are expressed as the mean ± standard deviation. Analysis of variance (ANOVA) was used for multiple comparisons of means, and the Tukey’s test and Student’s t-test were performed to substantiate differences between groups using Excel Statistics for Windows. The differences were considered statistically significant for *p* < 0.05.

## 3. Results

### 3.1. Extract Preparation and Characterization

The skins separated from pomace of the Carignano grape were pre-treated with distilled water to reduce the content of sugars and, in turn, the hygroscopicity and instability of the final extract. After freeze-drying, samples were ground to both obtain a fine powder composed of particles with a high specific surface area (mean diameter ~82 µm) and promote the interaction between particles and the extractive medium. Ultrasound assisted extraction was performed in a hydroethanolic solution (70:30 *v*/*v*) to ensure the extraction of polar and low polar molecules. The extraction yield was not high, as it was ~7%. The total phenolic content of the extract was 23.8 mg gallic acid equivalent/g of dry extract, and the antioxidant activity was 4.3 mg trolox equivalent/g of dry extract [18].

### 3.2. Vesicle Preparation and Characterization 

Grape pomace extract was incorporated at a high concentration (40 mg/mL) in liposomes and nutriosomes prepared by direct sonication, which is an environmentally-friendly method that avoids the use of pollutants’ organic solvents and is suitable for food additives [26]. Nutriose FM06^®^ was combined with soy lecithin to obtain nutriosomes whereas gelatin was added to reinforce vesicle resistance in the oral route and obtain gelatin-liposomes and gelatin-nutriosomes. 

**Figure 1:**
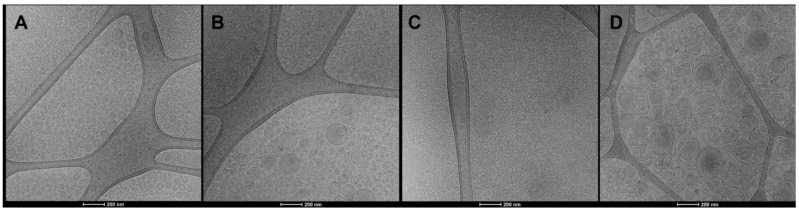
Representative cryo-TEM images of grape pomace extract loaded liposomes (**A**), nutriosomes (**B**), gelatin-liposomes (**C**) and gelatin-nutriosomes (**D**).

Cryo-TEM analyses confirmed the formation of closed and lamellar vesicles (Figure 1). Liposomes and gelatin-liposomes were small, spherical, unilamellar and mostly homogeneously dispersed (Figure 1A–C). The addition of Nutriose FM06^®^ improved lamellarity and roundness and allowed the assembling in peculiar multicompartment vesicles (Figure 1B,D), as previously reported [16]. Dynamic and electrophoretic light scattering measurements confirmed the formation of small (~102 nm) and monodispersed vesicles, being the polydispersity index ≤ 0.18 (Table 1).

Empty liposomes, nutriosomes and gelatin-liposomes were slightly smaller than the corresponding vesicles loading the extract (*p* < 0.05), indicating the intercalation of the components contained in the phytocomplex within the bilayer and the modification of its assembling, thus leading to the formation of larger vesicles with a lower curvature radius. Empty and extract loaded gelatin-nutriosomes had instead the same mean diameter (~105 nm, *p* > 0.05). Among the extract loaded vesicles, liposomes were the smallest (~94 nm, *p* < 0.05 versus extract loaded nutriosomes, gelatin-liposomes, gelatin-nutriosomes), and the addition of Nutriose FM06^®^ or gelatin or their combination led to the formation of slightly larger vesicles (~105 nm, *p* < 0.05 versus extract loaded liposomes). Regardless of the mean diameter, these systems remained highly monodispersed, as their polydispersity indexes were ≤0.17. All vesicles loaded high amounts of extract, as the entrapment efficiency was ~82% (*p* > 0.05 among the values of liposomes, nutriosomes, gelatin-liposomes and gelatin-nutriosomes), irrespective of the formulation tested. The zeta potential of the formulations was highly negative due to the negative group of phosphatidylcholines at the used pH (~5.5) [27]. This could improve the storage stability of vesicles in dispersion over time because it increases the electrostatic repulsion among vesicles, inhibiting their agglomeration and fusion [28]. 

### 3.3. Stability of Extract Loaded Vesicles in Fluids Simulating Oral, Gastric and Intestinal Environments

When administered orally, formulations are diluted in media, having different pH values and ionic strengths along the gastro-intestinal tract, which may cause degradation of carriers and payloads and result in an ineffective oral administration [10]. For this reason, vesicles were diluted and incubated with the appropriate medium mimicking the ones that can be found in the oral route. At first, the behavior of the vesicles in simulated saliva (pH 6.75) was assessed measuring the changes of their mean diameter, polydispersity index and zeta potential (Table 2). Vesicles enriched with gelatin were affected by salivary pH as the mean dimeter of gelatin-liposomes increased up to ~204 nm (*p* < 0.05 versus the initial value ~104 nm) and those of gelatin-nutriosomes up to ~206 nm (*p* < 0.05 versus the initial value ~108 nm). Along with this, the polydispersity index of both increased up to ~0.28 (*p* < 0.05 versus the initial value ~0.15), indicating the formation of larger vesicles but still homogeneously dispersed. However, liposomes and nutriosomes prepared without gelatin were even more affected by the simulated saliva as their size increased up to ~270 nm (*p* < 0.05 versus the initial values, liposomes ~94 nm and nutriosomes ~103) and the polydispersity index up to ~0.37 (*p* < 0.05 versus the initial value ~0.15 for both vesicles). The addition of gelatin seems to be a valid strategy for vesicle stabilization at salivary pH, probably due to its ability to promote intermolecular cross-bindings within the vesicles [29]. Moreover, considering that the zeta potential of all the vesicles increased to almost 0 mV, it can be concluded that the detected instability was mainly related to the surface charge neutralization at this pH (Table 2).

To further investigate the vesicles’ stabilities at the gastro-intestinal level, they were also incubated with a medium at pH 1.20 (mimicking the gastric pH) for 2 h or at pH 7.00 (mimicking the intestinal pH) for 6 h, and their main physico-chemical characteristics were measured (Table 2). Again, the incubation (t_2_) with the medium at pH 1.20 caused a significant increase of vesicles. Liposomes and nutriosomes were the most affected as the diameter of liposomes increased from ~94 nm to ~339 nm (*p* < 0.05 between the two values) and that of nutriosomes from ~103 nm to ~372 nm (*p* < 0.05 between the two values). On the contrary, gelatin-liposomes and gelatin-nutriosomes underwent a lesser enlargement as the mean diameter increased from ~106 nm to ~243 nm (*p* < 0.05, between the two values). The polydispersity index had a similar trend, as that of liposomes and nutriosomes increased, remaining similar to that of gelatin-liposomes whereas for gelatin-nutriosomes (~0.20), it remained almost unchanged. Similarly, to that observed with the simulated saliva, the zeta potential of all the tested vesicles was significantly affected by the pH, reaching almost 0 mV regardless of the vesicle composition (Table 2) due to the presence of salts in the medium [6]. The incubation with the medium at pH 7.00 also led to a significant increase of the mean diameter, and that of liposomes reached ~291 nm (*p* < 0.05 versus the initial value ~94 nm) and that of nutriosomes ~216 nm (*p* < 0.05 versus the initial value ~103 nm). The addition of gelatin to liposomes or nutriosomes seemed to effectively improve vesicle stability as in the case of the salivary pH, due to the close pH value. According to this, a moderate increase of the size and polydispersity index of gelatin-liposomes and gelatin-nutriosomes was observed and, from the initial value of ~106 nm, the mean diameter of the firsts arrived to ~196 nm whereas that of the formers to ~222 nm (*p* < 0.05 between the initial value and the corresponding values at 6 h and between the values of the two samples at 6 h). The enlargement was presumably related to the swelling of gelatin which led to the relaxation of chains and their entanglement to form an interconnected and stable system (Table 2) [29].

### 3.4. Biocompatibility of Vesicles

**Figure 2:**
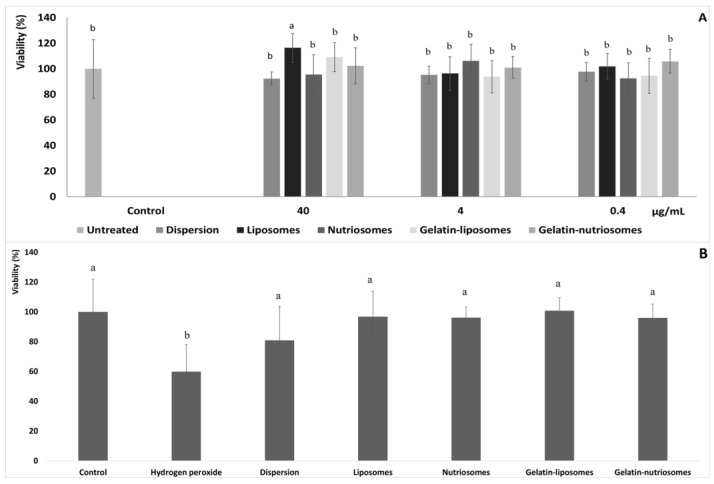
(**A**) Viability of Caco-2 cells incubated for 48 h with grape pomace extract in dispersion or loaded in liposomes, nutriosomes, gelatin-liposomes and gelatin-nutriosomes, diluted at different concentrations (40, 4 and 0.4 µg/mL). Mean values ± standard deviations were reported (n = 8). Same letter (a) indicates not statistically different values (*p* > 0.05 versus untreated cells) and statistically different from other (*p* < 0.05 versus other formulations). (**B**) Viability of Caco-2 cells stressed with hydrogen peroxide (1:40,000) and treated with grape pomace extract (4 µg/mL) in dispersions or loaded in liposomes, nutriosomes, gelatin-liposomes and gelatin-nutriosomes. Mean values ± standard deviations were reported (n = 8). Same letter (a and b) indicates not statistically different values (*p* > 0.05 versus untreated cells) and statistically different from other (*p* < 0.05 versus other formulations).

Human colon adenocarcinoma (Caco-2) cells, when grown on semi permeable filters, spontaneously differentiate in culture to form confluent monolayers which structurally and functionally resemble the small intestinal epithelium [30]. Aiming at evaluating the biocompatibility of vesicles, after differentiation to the epithelial cell monolayer, Caco-2 cells were chosen as representative cells of the intestinal epithelia and were treated for 48 h with the extract, in dispersion or loaded in vesicles, at three different dilutions (40, 4 and 0.4 µg/mL). Cell viability was ~100%, irrespective of the formulation and the concentration tested (*p* > 0.05 versus untreated cells). Only using the highest concentration of liposomes and gelatin-liposomes (40 µg/mL), the viability of cells increased up to ~116% (*p* < 0.05 versus other formulations) and ~109% (*p* > 0.05 versus other formulations). This confirmed the well-known high biocompatibility of the extract loaded in phospholipid vesicles (Figure 2A) [26].

### 3.5. Protective Effect of Vesicles against Damages Induced by Hydrogen Peroxide in Cells

The ability of extract loaded vesicles to protect intestinal cells from oxidative damage was also assessed and compared to that of the extract in aqueous dispersion. Considering the previous results and the high biocompatibility of vesicles at all the concentrations tested, 4 µg/mL was chosen as the non-cytotoxic concentration (Figure 2B).

The stress of Caco-2 with hydrogen peroxide (1:40,000 dilution) significantly affected the cells viability, which drastically decreased to ~60% (*p* < 0.05 versus untreated cells). The simultaneous treatment with the extract in the dispersion instead partially protected the cells from damages or death induced by hydrogen peroxide, as the viability was ~80% (*p* < 0.05 versus untreated cells). Lastly, the treatment with the extract loaded in the vesicles effectively counteracted or prevented the damages induced by the dangerous oxidative molecule, avoiding cell death, as the viability was always ~100% (*p* > 0.05 versus untreated cells), irrespective of the vesicle composition. These results agreed with the previous findings confirming the high antioxidant power of the molecules contained in the grape extract and the ability of vesicles to promote the beneficial properties of the loaded molecules [31].

### 3.6. Stability of Vesicles Mixed with Milk Whey

The stability of the vesicles mixed with milk whey at different concentrations (5, 10 and 20%) was evaluated to predict their possible addition to the yogurt. The mean diameter and polydispersity index were measured at 1 and 24 h of incubation at 4 °C (Table 3), which are the minimum and the maximum recommended storage temperatures for dairy products.

Milk whey was slightly acidic (pH 4.55) and contained particles sized ~298 nm (*p* < 0.05 versus other values, *p* > 0.05 versus the value of gelatin-nutriosomes at 24 h) and polydispersed, as the polydispersity index was ~0.38, according to results previously reported [32]. 

At 1 h of storage in whey, vesicles were maintained as almost unchanged in their structure, as the mean diameter of 5% and 10% of liposomes was ~118 nm (*p* < 0.05 between the two formulations) whereas at the highest concentration (20%), it was higher, ~136 nm (*p* < 0.05 versus other formulations). Other formulations, irrespective of the used concentration, had a similar behavior and the size of nutriosomes became ~128 nm (*p* < 0.05 versus initial value), that of gelatin-liposomes ~116 nm (*p* < 0.05 versus initial value) and that of gelatin-nutriosomes ~118 nm (*p* > 0.05 versus initial value). 

After 24 h of storage, the vesicle size changed significantly according to their composition and concentration in whey. An amount of 10 or 20% of liposomes and 20% of nutriosomes and gelatin-liposomes had an average diameter >1000 nm (*p* < 0.05 compared with the other values) and polydispersity indexes ~0.93, suggesting strong time-dependent interactions of vesicles with whey components. This effect has been described as a consequence of the interactions between serum proteins and lipids with phospholipids and is specifically addressed by Van der Waals and electrostatic forces that may lead to the breakdown of vesicles and the new assembly of their components into larger aggregates [33,34]. Differently, at 24 h, the size of 5% of liposomes and nutriosomes increased less, reaching ~353 nm (*p* > 0.05 between the two values), three times larger than the starting value (~104 nm *p* < 0.05 versus the other values and *p* > 0.05 between freshly prepared gelatin-nutriosomes) and the polydispersity index increased up to ~0.55. Nonetheless, at this concentration (5%), they seemed more to enlarge than break, as their size was still plausible. The enlargement of the 20% of gelatin-liposomes in whey instead can be attributed to electrostatic attractions between whey protein and gelatin, which are known to lead to vesicle fracturing [35]. These interactions appeared to be gelatin concentration-dependent, as they decreased when the concentration of vesicles in whey decreased. As a consequence, the size of 10% of gelatin-liposomes was ~636 nm (*p* < 0.05 versus other values) and that of 5% of them was ~297 nm (*p* < 0.05 versus other values except versus milk whey values). By contrast, gelatin-whey interactions were negligible after the addition of Nutriose FM06^®^ in vesicles, regardless of the concentration. According to other studies, this may be related to the ability of dextrin to mechanically coat the surface of the vesicles, preventing electrostatic interactions and reinforcing the structure of the vesicles, which are thus more stable and resistant [36,37]. In line with this, the mean diameter and polydispersity index of gelatin-nutriosomes in whey underwent only a slight increase (up to ~234 nm at 20%, ~207 nm at 10% and ~173 nm at 5%; *p* < 0.05 versus the other values). Results suggest that the combination of Nutriose FM06^®^ and gelatin improved the stability of the vesicles, especially upon dilution in whey. 

### 3.7. Viscosity Studies

**Figure 3:**
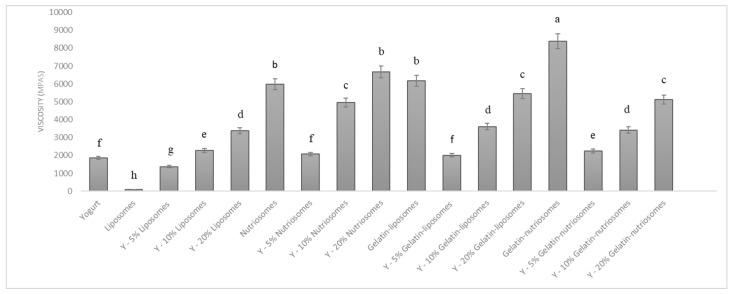
Viscosity values of yogurt containing different concentrations (0, 5, 10 and 20%) of liposomes, nutriosomes, gelatin-liposomes and gelatin-nutriosomes. Mean values ± standard deviations (error bars) were reported. Same letter (a, b, c, d, e, f, g and h) indicates not statistically different values (*p* > 0.05), while different letters indicates statistically different values (*p* < 0.05).

The vesicles were mixed with the yogurt at different concentrations (5, 10 and 20%) to obtain an enriched food. The changes in viscosity of yogurt samples, after the manual addition of dispersions, were evaluated at 25 °C (Figure 3). The viscosity of white yogurt was ~1900 mPas and that of liposomes, as expected, was lower, ~90 mPas (*p* < 0.05 versus yogurt). The addition of liposomes to the yogurt changed the viscosity as a function of the concentration, being ~1300 mPas (*p* > 0.05 versus viscosity of white yogurt; *p* < 0.05 versus that of liposomes) at 5%, 2600 mPas (*p* > 0.05 versus viscosity of white yogurt; *p* < 0.05 versus that of liposomes) at 10% and 3300 mPas (*p* < 0.05 versus viscosity of white yogurt; *p* < 0.01 versus that of liposomes) at 20%. Nutriosomes and gelatin-liposomes had a higher viscosity than those of liposomes and white yogurt, being ~5900 mPas (*p* > 0.05 between the viscosity of the two samples; *p* < 0.05 versus the white yogurt viscosity), due to the interactions and thickening effect of Nutriose FM06^®^ and gelatin in the media [38]. As expected, the addition of nutriosomes to the yogurt led to a linear increase of viscosity as a function of the used concentration and the viscosity was at 5% ~2000 mPas (*p* > 0.05 versus the white yogurt viscosity; *p* < 0.05 versus the viscosity of yogurt containing 10 or 20% nutriosomes); at 10% ~5500 mPas (*p* < 0.05 versus the white yogurt viscosity; *p* < 0.05 versus viscosity of nutriosomes); and at 20% ~6600 mPas (*p* < 0.01 versus the white yogurt viscosity; *p* > 0.05 versus viscosity of nutriosomes). The addition of gelatin-liposomes led to a similar behavior and the viscosity was at 5% ~2000 mPas (*p* > 0.05 versus the white yogurt viscosity; *p* < 0.05 versus the viscosity of gelatin-liposomes) and at 20%, reached ~5400 mPas (*p* < 0.05 versus the viscosity of gelatin-liposomes and white yogurt). Gelatin-nutriosomes had the highest viscosity: ~8300 mPas (*p* < 0.05 versus the viscosity of other vesicle dispersions and white yogurt); their addition to the yogurt affected the samples’ viscosity in a concentration-dependent manner but to a less extent than nutriosomes and gelatin-liposomes. Indeed, the viscosity of yogurt containing gelatin-nutriosomes at 5% was ~2200 (*p* > 0.05 versus the white yogurt viscosity; *p* > 0.05 versus the viscosity of gelatin-liposomes), and at 20% was ~5100 mPas (*p* < 0.05 versus the viscosity of gelatin-nutriosomes and white yogurt). 

## 4. Discussion

The consumption of dairy products is steadily increasing among the population and yogurt has been considered an excellent source of proteins, calcium, vitamins, lipids and minerals. It also contains lactose-fermenting bacteria that confer health benefits, reducing constipation and stimulating the immune system [39]. The addition of functional additives could give the product an extra edge, exploring new horizons on healthy and nutritional foods [40]. Polyphenols and antioxidant molecules or phytocomplexes are optimal candidates to be added to the yogurt, which exert complementary and synergic activities with the yogurt [41]. Their effectiveness could be strengthened by the loading in phospholipid vesicles tailored for oral administration [42]. To be added to foods such as yogurt, vesicles must be stable in milk whey and should not significantly modify the nutritional value, food safety and consumer acceptance of the final product [43]. Toniazzo et al. confirmed the suitability of functional yogurts enriched with β-carotene-loaded liposomes, which were homogeneous and did not separate after their addition [44]. According to this, in the present study, the grape pomace extract was used as an eco-sustainable and low-cost source of antioxidant molecules, and nutriosomes were selected as the most promising carriers for the oral delivery of natural molecules, while liposomes were prepared as a comparison [15]. Vesicles were prepared by direct sonication, an easy method that avoids the use of pollutants’ organic solvents and dissipative steps [26]. The extract was used at a high concentration (40 mg/mL) and so was soy lecithin (240 mg/mL), to ensure the stable incorporation of the components of the extract inside the vesicles. The size of the extract loaded vesicles was higher than that of empty ones, because of the intercalation of phytochemicals inside the bilayer. Luo et al. observed a similar trend, as the size of liposomes increased with increasing concentrations of procyanidins [45]. The addition of Nutriose FM06^®^, gelatin or their combination did not significantly modify vesicle assembling nor their zeta potential, which was highly negative, due to the negative group of phosphatidylcholines at the pH of formulations (~5.5) [27]. The vesicle charge may contribute to their stability in dispersion due to the strong repulsion forces among them, preventing aggregation or fusion phenomena [28]. Regardless of the composition, all vesicles had high entrapment efficiency (≥82%), in perfect agreement with previous results obtained with other natural molecules loaded in phospholipid vesicles [46]. Nutriose FM06^®^ and gelatin played a key role in improving the suitability of vesicles as a food additive. Indeed, gelatin-liposomes and even more gelatin-nutriosomes were less affected by the pH values of the oral route simulating salivary, gastric and intestinal environments (6.75, 1.20 and 7.00, respectively). The optimal resistance of nutriosomes at these pH values was previously confirmed and is likely due to its distribution inside the vesicles and on their surface, forming a protective layer. Nutriosomes’ performances were further improved by the gelatin, which allowed the formation of multicompartment vesicles [47]. Gelatin is a natural protein polymer widely used in pharmaceutical and food industries as a thickening agent, due to its biocompatibility, biodegradability and muco-adhesiveness [48]. It could form an additional layer on the vesicle surface, promoting the interactions among vesicles and preserving their structure [28]. The stability of vesicles in milk whey was optimal at 1 h as they maintained an almost unchanged structure, while at 24 h, only gelatin-nutriosomes were stable. This highlighted that gelatin-nutriosomes, thanks to their peculiar composition and structure, could be added extemporarily and used as a topping. Similarly, Molina et al. (2019) demonstrated that nanoparticles can be successfully added to yogurt just before consumption to avoid problems related to their instability in whey [49]. The addition of 10 and 20% of vesicle dispersions to the yogurt increased the viscosity of the final product that in turn may improve consumer enjoyability together with the nutritional quality of it, thanks to the addition of grape polyphenols and Nutriose FM06^®^. Indeed, polyphenols loaded in vesicles effectively protect the intestinal cells from damages caused by oxidative stress, and Nutriose as prebiotic favors for intestinal health [10]. These beneficial effects, along with those of the yogurt may promote the growth of gut microbiota, enhance the intestinal barrier status as well as maintain intestinal homeostasis and physiological functions such as the synthesis of vitamins and promotion of immune system maturation [50].

## 5. Conclusions

Overall results suggest the appropriateness of grape pomace extract loaded in gelatin-nutriosomes as a topping for yogurt. They are more stable in gastro-intestinal conditions and in milk whey, when added to the yogurt. Grape polyphenols loaded in vesicles protect the intestinal cells from oxidative stress caused by ROS and, in synergy with Nutriose FM06^®^ and yoghurt strains, contribute to intestinal homeostasis. Therefore, once added directly to yogurt as a functional topping just before the consumption of the dairy products, grape extract loaded gelatin-nutriosomes can complement their nutritional values, favoring a correct physiological function of the gut. These preliminary results will be better explored by means of in vivo studies using animal models and sensorial assays.

## Figures and Tables

**Table 1:** Mean diameter (MD), polydispersity index (PI), zeta potential (ZP), entrapment efficiency (EE) of empty and grape pomace extract loaded in liposomes, nutriosomes, gelatin-liposomes and gelatin-nutriosomes. Mean values ± standard deviations are reported (n ≥ 6). The same letter (**a, b, c, d, e**) indicates not statistically different values (*p* > 0.05 among values with same letter and *p* < 0.05 versus values with different letters).

	MD (nm)	PI (PI)	ZP (mV)	EE (%)
Empty liposomes	75 ± 4	0.19	^**d**^ - 48 ± 1	-
Empty nutriosomes	^**b**^ 90 ± 4	0.16	^**e**^ - 57 ± 3	-
Empty gelatin-liposomes	^**b**^ 92 ± 3	0.21	^**d,e**^ - 50 ± 7	-
Empty gelatin-nutriosomes	^**a**^ 103 ± 2	0.16	^**d,e**^ - 50 ± 7	-
Liposomes	^**b**^ 94 ± 3	0.18	^**d**^ - 48 ± 3	^**c**^ 84 ± 11
Nutriosomes	^**a**^ 103 ± 5	0.12	^**d,e**^ - 50 ± 5	^**c**^ 82 ± 7
Gelatin-liposomes	^**a**^ 104 ± 3	0.17	^**d**^ - 48 ± 3	^**c**^ 86 ± 7
Gelatin-nutriosomes	^**a**^ 108 ± 8	0.13	^**d**^ - 50 ± 2	^**c**^ 87 ± 13

**Table 2:** Mean diameter (MD), polydispersity index (PI) and zeta potential (ZP) of liposomes, nutriosomes, gelatin-liposomes and gelatin-nutriosomes diluted with medium at pH 6.75, 1.20 or 7.00 and high ionic strength (0.3 M sodium chloride), which mimic the saliva medium and gastro-intestinal environment. Mean values ± standard deviations are reported (n = 6). The same letter (**a, b, c, d, e, f, g**) indicates not statistically different values (*p* >  0.05 among values with same letter and *p* <  0.05 versus values with different letters).

	pH	Time	MD (nm)	PI	ZP (mV)
Liposomes	Freshly prepared		94 ± 3	0.18	^**g**^ - 48 ± 3
	6.75	t_10min_	^**a**^ 270 ± 10	0.37	^**f**^ - 2 ± 2
	1.20	t_2h_	339 ± 17	0.36	^**f**^ - 2 ± 2
	7.00	t_6h_	^**a**^ 291 ± 9	0.30	^**f**^ 0 ± 0
Nutriosomes	Freshly prepared		^**e**^ 103 ± 5	0.12	^**g**^ - 50 ± 5
	6.75	t_10min_	^**a,b**^ 266 ± 21	0.36	^**f**^ - 1 ± 3
	1.2	t_2h_	372 ± 11	0.32	^**f**^ 0 ± 1
	7	t_6h_	^**d**^ 216 ± 5	0.31	^**f**^ 0 ± 2
Gelatin-liposomes	Freshly prepared		^**e**^ 104 ± 3	0.17	^**g**^ - 48 ± 3
	6.75	t_10min_	^**d**^ 204 ± 6	0.27	^**f**^ - 1 ± 1
	1.2	t_2_	^**b**^ 243 ± 10	0.18	^**f**^ 0 ± 0
	7	t_6_	^**d**^ 196 ± 8	0.22	^**f**^ 0 ± 3
Gelatin-nutriosomes	Freshly prepared		^**e**^ 108 ± 8	0.13	^**g**^ - 50 ± 2
	6.75	t_10min_	^**c,d**^ 209 ± 14	0.28	^**f**^ - 1 ± 2
	1.2	t_2_	^**b**^ 243 ± 9	0.22	^**f**^ - 1 ± 2
	7	t_6_	^**b,c**^ 222 ± 11	0.24	^**f**^ 0 ± 2

**Table 3:** Mean diameter (MD) and polydispersity index (PI) of liposomes, nutriosomes, gelatin-liposomes and gelatin-nutriosomes mixed with milk whey (pH 4.55) at different concentrations (5, 10 and 20%), stored at 4 °C for 1 h (t_1_) and 24 h (t_24_). Mean values ± standard deviations are reported (n = 3). The same letter indicates not statistically different values (**a, b, c, d, e, f, g, h**) (*p* >  0.05 versus values with same letter and *p* < 0.05 versus values with different letters).

	Concentration (% *v*/*v*)	Time	MD (nm)	PI
**Milk whey**			^**d**^ 298 ± 2	0.38
**Liposomes**		*Freshly prepared*	94 ± 3	0.18
	5	*t_1_*	^**g,f**^ 118 ± 2	0.22
		*t_24_*	^**c**^ 348 ± 9	0.63
	10	*t_1_*	^**g,f**^ 118 ± 2	0.20
		*t_24_*	^**a**^ 1113 ± 43	0.84
	20	*t_1_*	136 ± 1	0.24
		*t_24_*	^**a**^ 1498 ± 268	0.96
**Nutriosomes**		*Freshly prepared*	^**h**^ 103 ± 5	0.12
	5	*t_1_*	^**g**^ 112 ± 2	0.17
		*t_24_*	^**c**^ 356 ± 6	0.56
	10	*t_1_*	145 ± 1	0.23
		*t_24_*	^**b**^ 576 ± 52	0.80
	20	*t_1_*	^**e**^ 129 ± 2	0.22
		*t_24_*	3485 ± 10	1.00
**Gelatin-liposomes**		*Freshly prepared*	^**h**^ 104 ± 3	0.17
	5	*t_1_*	^**g,f**^ 118 ± 2	0.23
		*t_24_*	^**d**^ 297 ± 4	0.60
	10	*t_1_*	^**g,f**^ 116 ± 1	0.19
		*t_24_*	^**b**^ 636 ± 34	0.45
	20	*t_1_*	^**g,f**^ 115 ± 2	0.17
		*t_24_*	^**a**^ 1226 ± 110	0.93
**Gelatin-nutriosomes**		*Freshly prepared*	^**h,g**^ 108 ± 8	0.13
	5	*t_1_*	^**e**^ 124 ± 2	0.16
		*t_24_*	173 ± 5	0.30
	10	*t_1_*	^**g,f**^ 115 ± 1	0.11
		*t_24_*	207 ± 2	0.38
	20	*t_1_*	^**g,f**^ 117 ± 1	0.12
		*t_24_*	234 ± 10	0.39
